# PReS-FINAL-2174: The performance of the new slicc criteria for the classification of sle in children

**DOI:** 10.1186/1546-0096-11-S2-O9

**Published:** 2013-12-05

**Authors:** E Sag, A Ravelli, ED Batu, A Tartaglione, AS Khalil, S Marks, S Ozen

**Affiliations:** 1 Department of Pediatrics, Hacettepe University Faculty of Medicine, Ankara, Turkey; 2 Department of Pediatric Rheumatology, University of Genova, Genova, Italy; 3 Pediatric Nephrology, Great Ormond Street Hospital, London, UK; 4 Department of Pediatric Rheumatology, Hacettepe University Faculty of Medicine, Ankara, Turkey

## Introduction

The Systemic Lupus International Collaborating Clinics (SLICC) have recently suggested a new set of criteria for the classification of SLE. However the differences between sensitivity and specificities of the ACR criteria and the new SLICC criteria among pediatric SLE patients have not been investigated yet.

## Objectives

We aimed to compare the sensitivity and specificities of the ACR criteria and the new SLICC criteria among pediatric SLE patients.

## Methods

Three main lupus centers from Europe were included in this study. One of these centers was mainly a pediatric nephrology center from UK whereas one was a pediatric rheumatology center from Italy and the last one was a joint one from Turkey. Features present at onset in childhood-onset SLE (cSLE) patients, diagnosed and followed by these three departments between January 2000 to December 2012 were retrospectively analyzed. For the specificity analysis, patients admitted to the respective departments, in whom ANA was deemed necessary by the caring physician in the diagnostic work-up were included as controls. PASW 18,0 for Windows was used for statistical analysis.

## Results

Both criteria were analyzed in 154 cSLE patients with a mean age at disease onset of 12,7 years and 95 controls with a mean age of 8,6 years. In the overall group, the sensitivity and specificity of the ACR criteria were 76,6% and 91,6% respectively and that of the SLICC criteria were 98,7% and 82,1% respectively. Four hemolytic uremic syndrome (HUS) patients and four juvenile dermatomyositis (JDM) patients met the SLICC criteria whereas 22 lupus nephritis fell to meet the ACR criteria.

Between the three centers there were marked differences among certain clinical features. On the other hand when we compared our results with the reported prevalances of the criteria in adults, renal involvement, neurologic findings, hemolytic anemia, positive titers for ANA and anti-dsDNA were more frequent among children whereas chronic skin lesions were less (p < 0,005).

## Conclusion

In this pediatric cohort SLICC criteria performed better, was more sensitive (p < 0,001), had fewer misclassifications, however was less specific (p = 0,016). The specificity of the SLICC criteria was jeopardized with the HUS and JDM cases. The prevalance of certain criteria were significantly different between adults and children, this may necessitate further revision in pediatrics.

## Disclosure of interest

None declared.

